# On-Line NIR to Regulate Pervaporation Process: Application for Dehydration

**DOI:** 10.3390/membranes8030074

**Published:** 2018-09-04

**Authors:** Thomas La Rocca, Emilie Carretier, Thomas Clair, Martial Etienne, Philippe Moulin

**Affiliations:** 1 Aix-Marseille Université, CNRS, Centrale Marseille, M2P2 UMR 7340, Equipe Procédés Membranaires (EPM), Europôle de l’Arbois, BP80, Pavillon Laennec, Hall C, 13545 Aix-en-Provence CEDEX, France; thomas.LA-ROCCA@univ-amu.fr (T.L.R.); emilie.carretier@univ-amu.fr (E.C.); 2 SANOFI, Chemin de Meteline, 04200 Sisteron, France; Thomas.Clair@sanofi.com (T.C.); Martial.Etienne@sanofi.com (M.E.)

**Keywords:** on-line near-infrared spectroscopy, pervaporation, HybSi^®^ membrane, organic purification

## Abstract

The regeneration of volatile organic solvents via dehydration tests, from 90 wt %, was evaluated by pervaporation using an on-line near-infrared (NIR) spectrometer. Experiments were performed using a bis(triethoxysilyl)methane (BTESM)-based ceramic HybSi^®^ membrane at temperatures of 20, 30 and 40 °C. The presence of an on-line NIR allows continuous monitoring of the process without sampling, and quickly estimates mass fractions of species in the retentate. Dehydration tests were performed at 30 °C in order to confirm the on-line NIR reproducibility, and closely matched results obtained with an off-line densimeter. These results validated the usefulness of the on-line NIR and provided the same precision whatever the mass fraction in the retentate. A good on-line reproducibility was found, with an agreement between the on-line NIR and off-line densimeter, obtaining an average deviation of ±0.058 wt %, ±0.17 wt % and ±0.049 wt %, respectively, at 20, 30 and 40 °C.

## 1. Introduction

Organic compounds are commonly used as extraction, or reaction solvents, in the industrial field due to their physicochemical properties. They are mainly employed in the petrochemical, pharmaceutical, and food industries. Chlorinated (dichloromethane, trichloromethane) [1] and aromatic (benzene, cyclohexane) [2] solvents are among the most harmful and cause serious health problems. Because of their volatility and toxicity, as well as the introduction of more and more stringent legislation, it is necessary to set up new, suitable treatment processes in order to reduce their introduction into the environment, which may result in smog formation, and the damage of aquatic fauna and flora [3,4,5].

The use of regenerative processes is in line with better protection of the environment. Among these, pervaporation is being used more and more for purification and reuse, thanks to its economic advantages [6]. Indeed, this process, based on the differences in solubility and diffusivity of minority compounds through a non-porous membrane, across a partial pressure gradient, is suitable to separate azeotropes, which is not possible with distillation and it is less energy consuming. Also, pervaporation is usually associated with distillation in order to optimize the efficiency and energy consumption [7,8,9]. Other processes, such as liquid-liquid extraction, adsorption or air stripping, have also been applied but show several drawbacks. Extraction requires the presence of additional solvent often toxic [4] and adsorption requires a porous adsorbent, as well as implementation of a regeneration step [10,11]. Air stripping is only efficient for the extraction of very volatile compounds and can transform the water pollution into air pollution with frequent risks of fouling [10,11]. On an industrial scale, pervaporation is extensively used for dehydration, notably those of alcohols such as ethanol and isopropanol [12,13,14], but also for the VOC extraction [15,16,17] such as chlorinated compounds (dichloromethane, trichloroethylene) [1,18,19], toluene [20], benzene [2], or methyl-tert-butyl-ether (MTBE) [11]. 

In the conventional use of pervaporation, sampling is necessary to quantify permeation data, and measurements are usually analyzed by an off-line gas chromatograph. Regardless of its high precision, this analysis technique requires extensive handling of samples. Other alternatives were tested by Yeom et al. [21] using a system intermittently redirecting a part of the vapor permeate stream to a gas chromatograph (GC) to determine flow and permeate composition. This on-line device ensured a partial reduction of the sampling. Results show good agreement between steady-state flow measured with the on-line system, and the conventional method by liquid nitrogen, with a difference of ±2 g m^−2^ h^−1^. However, an expensive carrier gas was required for sample transfer.

Recently, some authors have focused their research on the use of on-line mass spectrometry (MS) acquisition for pervaporation due to its high sensitivity. MS is based on the measurement of the intrinsic molecular mass to charge ratio (m/z). Fraga et al. [22] performed dehydration tests of isopropanol and extraction tests of ethyl acetate, through a HybSi^®^ membrane, and a composite membrane of polyoctylmethylsiloxane on polyetherimide (POMS-PEI) respectively. This on-line system is used to better understand the mass transfer through membranes from the solution-diffusion model. The on-line MS made it possible to obtain permeation values, such as the composition of permeate streams and the diffusion coefficient (D), in the short time interval of 2 s. The on-line MS presents a real advantage, since it allowed the authors to observe that the diffusion coefficient stability of water in the HybSi membrane was faster than ethyl acetate in POMS, due to the more rigid structure of the HybSi^®^ membrane. A better analysis of the limitations of mass transport from steady-state conditions could therefore be incurred. Schäfer et al. [23] studied, with the same membrane, the case of aroma compounds (ethyl acetate, ethyl hexanoate) recovery from aqueous mixtures. The authors experienced the same benefits, and confirmed a good MS-signal reproducibility with a low variation of baseline, about 0.4% of the average value measured. The same experiments with multi-component mixtures were performed by Brazinha et al. [24] with an acquisition interval of every 12–15 s for partial pressures of water. Results show a deviation lower than 6% between data measured by a pressure gauge, and on-line MS. Therefore, the on-line MS improves the monitoring of pervaporation systems, but also requires a rigorous sampling protocol, which consumes the sample [24]. Toudji et al. [25] also used a new method to continuously measure mass flow applied to single component permeation such as water and ethanol. This new setup can be useful when applied to laboratory studies dealing with the evolution of mass flow according to different parameters of the process, for example, the permeate side pressure level or temperature of liquid feed.

In this work, on-line monitoring of the process was operated by near-infrared spectroscopy (NIR) acquisition. This analysis method, non-destructive and not needing a specific packaging for the sampling, was tested for acetone dehydration, from 90 wt %, through a BTESM-based ceramic HybSi^®^ membrane. These experiments were performed in the context of solvent regeneration in the pharmaceutical field. NIR spectroscopy is a qualitative and quantitative optical method based on the vibrational properties of molecules and their interactions with light at a given wavelength. The spectral range in NIR is between 0.78 and 2.5 µm [26,27] (approximately between 4000 and 13,000 cm^−1^). In this study, the use of on-line NIR allows for continuous operation, with an estimation of water contents in the retentate every few minutes without sampling. The on-line system allowed a better analysis of the effect of operating temperature on the dehydration efficiency. At first, flow and separation factor data were evaluated as a function of the water quantity in the retentate. Simultaneously, the evolution of water content was followed precisely and quickly by the on-line NIR and permitted real-time adjustment of the process. Results were compared at different temperatures. Then, the reproducibility of on-line NIR was tested at 30 °C, and a comparison was established between values of mass fraction from on-line NIR and off-line densimeter at 20, 30 and 40 °C.

## 2. Materials and Methods

### 2.1. Chemical Products and Membranes

Acetone was purchased at Sigma-Aldrich with a purity of 99.5 wt %. The water used was previously demineralized. The membrane was a hydrophilic HybSi^®^ membrane, described by Van Veel et al. [28] and Paradis et al. [29], and was supplied by Céramiques Techniques Industrielles (Salindres, France). Its structure is a hybrid with a ceramic support, an inner layer containing an amorphous silica, and an organic top layer made with bis(triethoxysilyl) methane (BTESM) on which zirconium (Zr) nanoparticles were grafted. The membrane was tube-shaped with seven channels and measured 1.2 m long for an active area of 0.15 m^2^. HybSi^®^ membranes are used mainly for the alcohol dehydration [4,30,31].

### 2.2. Experimental Set-up

**Figure 1:**
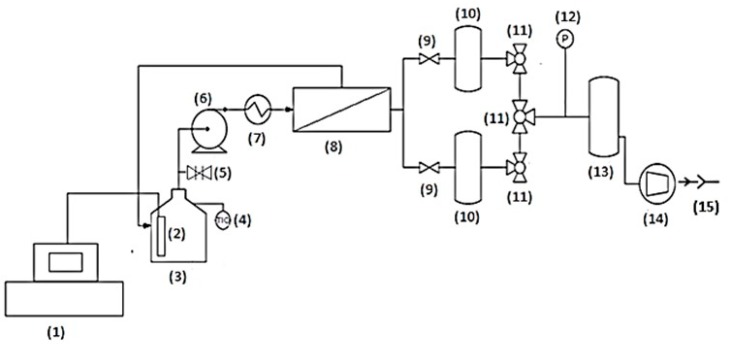
Pervaporation process set-up: (**1**) NIR Spectrometer with an acquisition software; (**2**) NIR probe; (**3**) Feed tank (5 L); (**4**) Temperature sensor (±0.2 °C from −30 to 120 °C); (**5**) Filing valve; (**6**) Volumetric pump (400 L h^−1^); (**7**) Heat exchanger; (**8**) Membrane; (**9**) Simple valve; (**10**) Cold traps with liquid nitrogen (1 L); (**11**) 3-way valves; (**12**) Vacuum pump sensor (< ±10% from 10^−2^ to 20 mbar); (**13**) Fail-safe cold trap (1 L); (**14**) Vacuum pump; (**15**) Air exhaust.

The pervaporation set-up is schematically represented in Figure 1. The NIR equipment includes a Fourier Transformed Infrared Spectrometer (IRTF) (11), operated with acquisition OPUS software , and a probe (2) with an optical path of 300 µm directly immersed in the liquid feed. This NIR package was purchased at Bruker France (Marne la Vallée, France). The 5 L feed tank (3) includes a temperature sensor (Hanna Instruments, Lingolsheim, France) (4). The liquid feed is fed to the membrane (8) by a volumetric pump (400 L h^−1^) (6) where its temperature is simultaneously regulated by a heat exchanger (Serflam, Vitrolles, France) (7), using ethylene glycol as a heat transfer fluid. A downstream (permeate side) pressure, below 3 mbar, is maintained by a vacuum pump (ATB, Vienna, Austria) (14) in order to ensure the required driving force for component permeation. A vacuum pressure sensor (Thyracont VD84, Passau, Germany) (12) provides monitoring of the applied downstream pressure. Permeate is condensed and collected periodically in cold traps (1 L) (10) cooled with liquid nitrogen at −196 °C. The system contains three traps: two operating in series (one trap and a fail-safe trap) and one, waiting and fitted, in parallel. The presence of a fail-safe cold trap (13) protects the vacuum pump against the last liquid drops from the condensate. Switching between the two traps connected in parallel is performed with the action of the valves (9,11).

### 2.3. Near-Infrared Spectroscopy Monitoring

**Figure 2:**
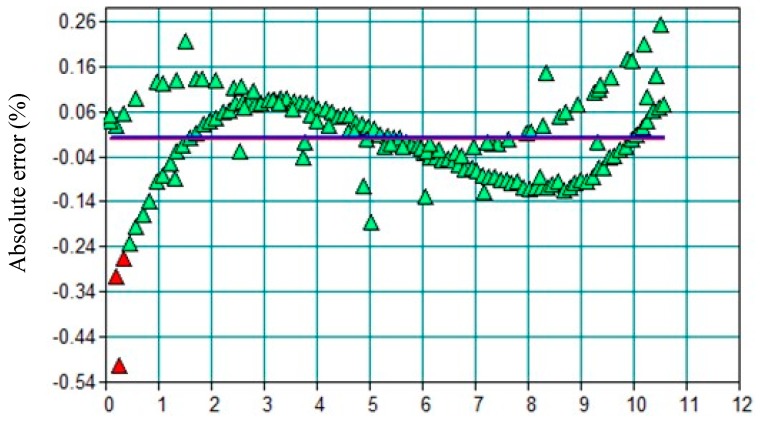
Absolute error vs weighed mass fraction of water for the NIR calibration.

The NIR probe was calibrated to the range of water content and temperature studied, namely from 0 to 10 wt % and from 20 to 40 °C, respectively. The spectral range of water was investigated between 7241.6 to 6695.9 cm^−1^. Calibration was performed at a steady temperature and after the homogenization of the acetone/water mixture, and showed a good correlation with an average absolute error below 0.1% (Figure 2). The horizontal line represents the zero reference point of absolute error. The monitoring of mass contents was carried out with 64 scan min^−1^.

### 2.4. Methods

Sampling at the permeate side was performed when the cold trap was recovered, so between 30 and 60 min. Sampling at the feed/retentate side was done in the same time interval and was analyzed by a densimeter (Anton Paar, Graz, Austria) with a precision of ±10 min^−6^ g cm^−3^, which was also calibrated to between 0 and 10 wt % water from an acetone-water mixture. To quantify the performance of the pervaporation process, the total flow of permeate was calculated after each switching of traps. Sampling in the feed and permeate side allows calculating the separation factor α [32]:

(1) αi,j=wp,iwp,jwf,iwf,j

where w represents the mass content of compounds i or j (%) and subscripts p and f denote the permeate and feed (retentate during the permeation) side, respectively.

### 2.5. Operational Conditions

Four acetone/water mixtures were prepared for dehydration tests at 20, 30 and 40 °C. Specifically, two mixtures were used to verify the on-line NIR reproducibility at 30 °C. Initial operational conditions for each experiment are listed in Table 1.

To analyze the effect of temperature on dehydration performance at the same operational conditions, similar masses were introduced in the feed for all experiments.

## 3. Results and Discussion

### 3.1. Effect of Operating Temperature

**Figure 3:**
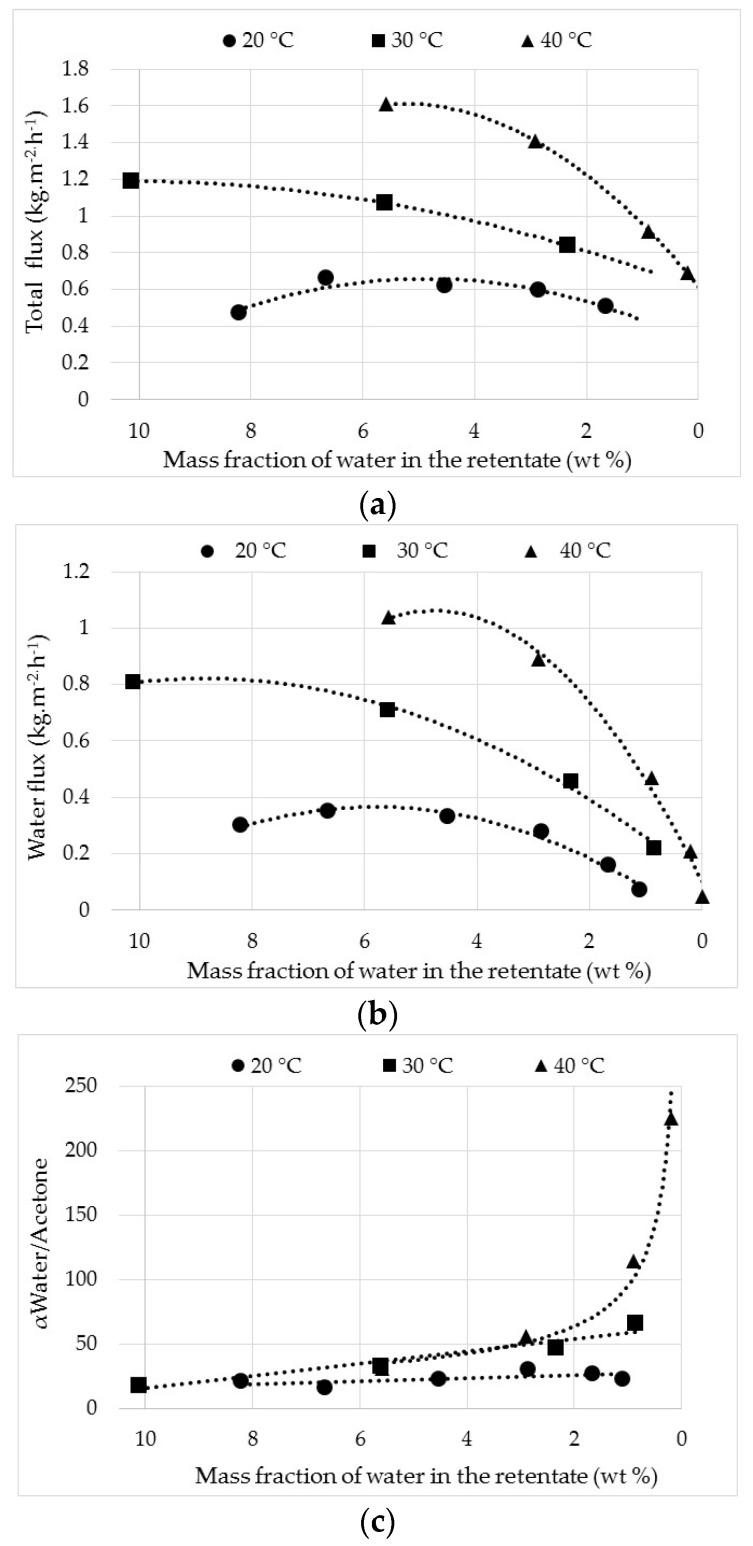
Evolution of total (**a**), water flow (**b**), and separation factor (**c**) from a binary acetone-water mixture (90/10 wt %) through the HybSi^®^ membrane depending on the water content in the retentate at 20, 30 and 40 °C.

The effect of operating temperature was studied at 20, 30 and 40 °C for permeate flow and separation factor. The evolution of these parameters, depending on the mass fraction of water in the retentate for each temperature, is illustrated in Figure 3.

Flow was calculated with a precision of ±0.1 g m^−2^ h^−1^, and water content was measured by the on-line NIR. The latter allows the visualization of the effect of water content on flow. The total (Figure 3a) and water flow (Figure 3b) decrease when the mass fraction of water decreases, whereas the acetone flow is not influenced by the decrease of water content and remains relatively constant around 0.3, 0.38 and 0.5 kg m^−2^ h^−1^ at 20, 30 and −40 °C, respectively. The variation of total and water flow follows the same trend because water is the preferential compound. The increase of temperature produces an increase of all initial flows, and the total and water flows decrease faster during dehydration when the temperature rises from 20 to 40 °C. Water extraction is therefore more efficient when the temperature increases. For each temperature, the water flow decreases to zero when dehydration occurs. The evolution of separation factor dependent on the mass fraction of water is given in Figure 3c. At 20 and 30 °C, the separation factor slowly increases from 16 to 30 and 18 to 66 respectively. Thus, these lower values confirm a dehydration performance less important at these two temperatures, notably at 20 °C. At 40 °C, the separation factor rapidly increases to 225 when the mass fraction of water moves towards 0 wt %. In this case, these higher values illustrate a strong performance of dehydration. The introduction of Zr nanoparticles into the BTESM could cause a more important affinity with water and improves consequently both flow and separation factor. A mass balance was performed in order to validate data.

The increase of flow conforms with the law of Arrhenius, as detailed by Luis and Van der Bruggen [33]. The increase of separation factor is made possible due to the rigid structure of the HybSi^®^ membrane, particularly its ceramic support. This structure limits the phenomenon of swelling of the membrane which involves an increase of free volume and consequently the permeation of both preferential and non-preferential compounds at the expense of separation factor. The free volume theory was revisited by Vrentas and Duda [34] for the diffusion phenomena in polymers. Swelling notably affects the structure of organic membranes which are more flexible than those of inorganic membranes. In this case, the swelling is not a factor and the increase of temperature facilitates the transport of smaller water molecules, improving the separation factor.

### 3.2. On-Line Nir Control

**Figure 4:**
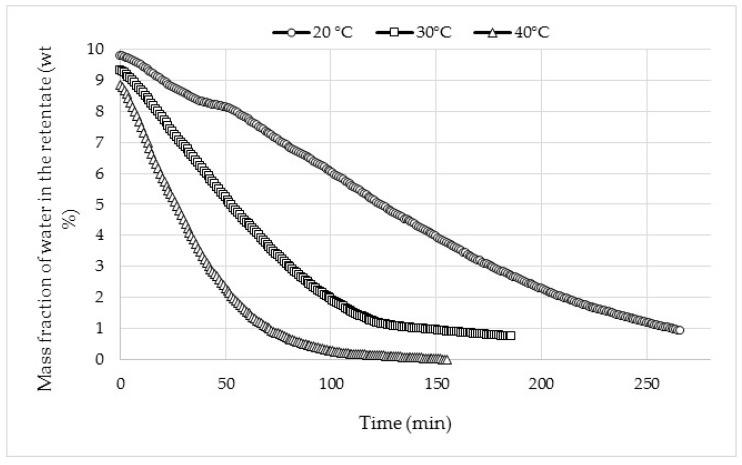
On-line monitoring of water content in the retentate during the acetone dehydration through the HybSi^®^ membrane depending on operating temperature.

The efficiency of dehydration at different temperatures was also evaluated by continuously recording the variation of tank composition versus time of dehydration (Figure 4).

In this case, the on-line NIR allows following, minute by minute, the evolution of water content during the dehydration even for very low concentrations of water. Moreover, the precision remains the same for both high and very low water contents regardless of the temperature. The analysis by on-line NIR is therefore perfectly adapted to high-precision control of the pervaporation process. The on-line NIR provides better observation of the positive effect of temperature on dehydration performance. Water content decreases much faster at 40 °C and a total recovery of water is observed, whereas at 20 and 30 °C, it seems to settle at around 0.93 and 0. 73 wt %, respectively. Dehydration performances regulated by the on-line NIR for each temperature are summarized in Table 2.

**Figure 5:**
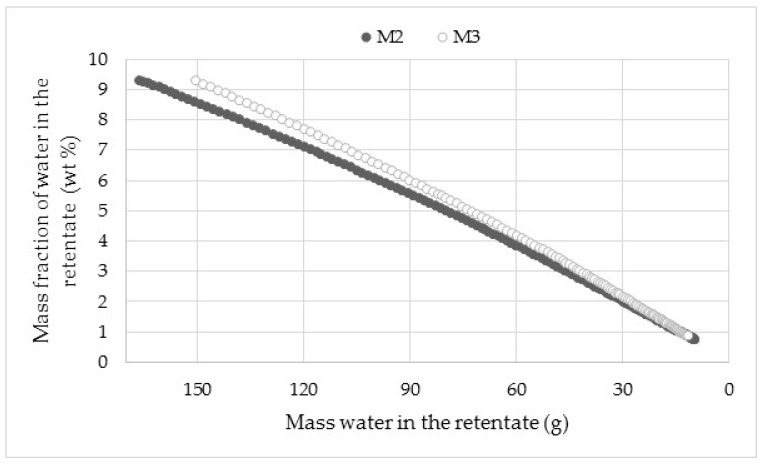
Reproducibility of on-line monitoring of acetone content versus mass water in the retentate through the HybSi^®^ membrane at 30 °C.

To validate the on-line NIR reproducibly, two dehydration tests (M2 and M3) were performed at 30 °C (Figure 5).

**Figure 6:**
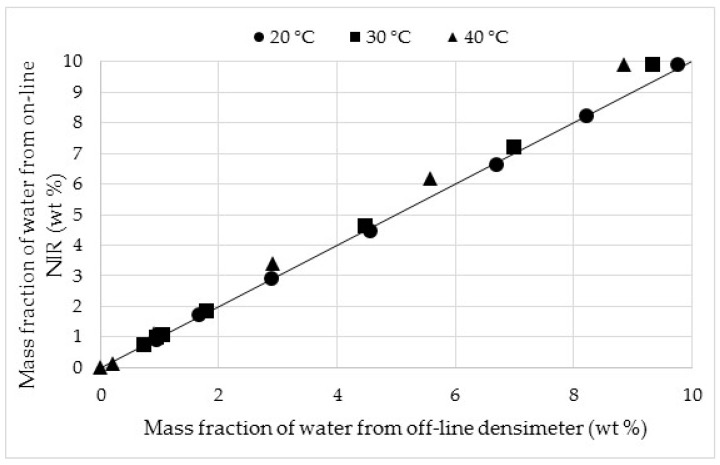
Comparison of measurements of water content from on-line NIR and off-line densimeter depending the temperature.

The on-line NIR shows a satisfactory reproducibility between M2 and M3. The gap gradually decreases towards 0 at low mass water in the retentate. The small variation in the first minutes of dehydration, when the mass water is high, could come from the temperature change. The latter has not yet reached the steady state notably at the beginning of experiment due to the influence of temperature polarization as Karlsson and Träghård [35] suggest. A local cooling from the feed stream is visible because of the energy of vaporization required for permeation compounds. In this case, the phenomenon causes a slight drop in the feed temperature of 2–3 °C and complicates the establishment of the steady state of the temperature. Reproducibility experiments were performed only at 30 °C, because the NIR equipment was already in use for other industrial chemical processes (distillation, etc.), to measure each intermediate reaction before proceeding to the next step. For all these utilizations, a good reproducibility was obtained for the application to pervaporation. The on-line NIR measurements of mass fraction of water were compared with the off-line densimeter to estimate the on-line acquisition reliability at different operating temperatures. This comparison is illustrated in Figure 6.

A good concurrence was found whatever the temperature. The average standard deviations at 20 and 30 °C were ±0.058 and ±0.17 wt %, respectively. At 40 °C, an average value of ±0.49 wt % was observed, and this higher value can be explained by a slight evaporation/condensation of acetone in the feed tank. Thus, the on-line NIR showed a very high reliability at each temperature and confirms its strong adaptability to the control of pervaporation processes.

## 4. Conclusions

In this work, on-line monitoring by NIR spectroscopy was tested during acetone dehydration by pervaporation through a HybSi^®^ membrane. The on-line NIR acquisition represents a real economic opportunity in the control of the pervaporation process. This on-line system reduces the sampling and therefore involves an important time saving, along with allowing real-time adjustment of the process. Additionally, it offers improved security and hygienic conditions. Results confirmed that the online monitoring facilitated the evaluation of the effect of temperature on dehydration performance. Therefore, it is possible to follow, with a high level of accuracy, the evolution of water contents even at very low levels, and ways to improve industrial performance. The on-line NIR reproducibility was validated by comparing measurements at 30 °C, where the minor gap might have an origin in the polarization temperature phenomena. Additionally, good agreement was observed for values of water contents measured by the on-line NIR and off-line densimeter, whatever the temperature, with a maximal standard deviation of ±0.49 wt % at 40 °C. The same NIR equipment has already been integrated at the industrial scale and has proven its reproducibility for VOC dehydration, namely tetrahydrofuran (THF), in the distillation process. The next research will focus on the use of on-line NIR in pervaporation on an industrial scale [36] for more complex solutions, and will be the subject of forthcoming papers. For this purpose, preliminary organic dehydration tests such as butan-1-ol and THF, with an off-line densimeter, were performed on a semi-industrial pilot of pervaporation and has shown very satisfactory results.

## Figures and Tables

**Table 1:** Summary of initial operational conditions for acetone dehydration through the HybSi^®^ membrane.

Mixture	Mass Fraction of Water (wt %)	Water Mass (kg)	Temperature (°C)
M1	9.8	0.19	20
M2	9.3	0.18	30
M3	9.4	0.16	30
M4	8.8	0.17	40

**Table 2:** Summary of dehydration performances of acetone at 20, 30 and 40 °C through the HybSi^®^ membrane.

Mixture	Initial/Final Mass Fraction of Water (wt %)	Purification Time (min)	Temperature (°C)
M1	9.8/0.93	265	20
M2	9.3/0.73	185	30
M4	8.8/0	155	40
